# Evaluation of ADAM-12 as a Diagnostic Biomarker of Ectopic Pregnancy in Women with a Pregnancy of Unknown Location

**DOI:** 10.1371/journal.pone.0041442

**Published:** 2012-08-21

**Authors:** Andrew W. Horne, Jeremy K. Brown, Stephen Tong, Tu'uhevaha Kaitu'u-Lino

**Affiliations:** 1 MRC Centre for Reproductive Health, University of Edinburgh, Queen's Medical Research Institute, Edinburgh , United Kingdom; 2 Translational Obstetrics Group, Mercy Hospital for Women, Heidelberg, Australia; John Hunter Hospital, Australia

## Abstract

**Background:**

Ectopic pregnancy (EP) remains the most life-threatening acute condition in modern gynaecology. It remains difficult to diagnose early and accurately. Women often present at emergency departments in early pregnancy with a ‘pregnancy of unknown location’ (PUL) and diagnosis/exclusion of EP is challenging due to a lack of reliable biomarkers. Recent studies suggest that serum levels of a disintegrin and metalloprotease protein-12 (ADAM-12) can be used differentiate EP from viable intrauterine pregnancy (VIUP). Here we describe a prospective study evaluating the performance of ADAM-12 in differentiating EP from the full spectrum of alternative PUL outcomes in an independent patient cohort.

**Methodology/Principal Findings:**

Sera were collected from 120 patients at their first clinical presentation with a PUL and assayed for ADAM-12 by ELISA. Patients were categorized according to final pregnancy outcomes. Serum ADAM-12 concentrations were increased in women with histologically-confirmed EP (median 442 pg/mL; 25%–75% percentile 232–783 pg/mL) compared to women with VIUP (256 pg/mL; 168–442 pg/mL) or miscarriage (192 pg/mL; 133–476 pg/mL). Serum ADAM-12 did not differentiate histologically-confirmed EP from spontaneously resolving PUL (srPUL) (416 pg/mL; 154–608 pg/mL). The diagnostic potential of ADAM-12 was only significant when ‘ambiguous’ PUL outcomes were excluded from the analysis (AROC = 0.6633; P = 0.03901).

**Conclusions/Significance:**

When measured in isolation, ADAM-12 levels had limited value as a diagnostic biomarker for EP in our patient cohort. The development of a reliable serum biomarker-based test for EP remains an ongoing challenge.

## Introduction

The diagnosis of ectopic pregnancy (EP) continues to present a major clinical challenge in obstetrics and gynecology, with patients often asymptomatic or presenting with non-specific symptoms that do not readily differentiate EP from miscarriage or viable intrauterine pregnancy.

Whilst in many cases, an EP will be detected by transvaginal ultrasonography (TVUSS) at the first clinic visit [1], TVUSS is often inconclusive and the pregnancy has to be initially classified as a “pregnancy of unknown location” (PUL) [2]. In patients with a PUL, subsequent diagnosis of EP relies on the serial measurement of serum human chorionic gonadotrophin (hCG) levels (and, in some centers, progesterone), together with follow-up TVUSS [3]–[5]. This approach significantly delays the diagnosis and management of EP and is resource intense and expensive [6]. There remains an unmet clinical need for a serum biomarker capable of identifying EP at first clinical presentation [5], [7].

Recently, Rausch et al [8] found a statistically significant decrease in a disintegrin and metalloprotease protein-12 (ADAM-12) in the sera of patients with EP (median 2.5 ng/mL), when compared to women with viable intrauterine pregnancy (median 18.6 ng/mL). The authors demonstrated this difference in a large cohort of 199 patients in the United States presenting with pain or bleeding in the first trimester of pregnancy. There appeared to be good discrimination between the groups as assessed by receiver operating characteristics (Area under ROC curve = 0.82; P<0.0001). They concluded that serum ADAM-12 was a promising biomarker for the diagnosis of ectopic pregnancy in women with symptoms in the first trimester.

However, there is debate as to the specificity of ADAM-12 with regard to differentiating EP from outcomes other than VIUP [9] due to the fact that other conditions, such as trisomy 21 can also present with alteration of ADAM-12 [10], [11]. Furthermore, the promising findings reported by Rausch et al needed independent verification. We therefore set out to validate Rausch et al's findings, measuring ADAM-12 in a cohort of women prospectively recruited in the United Kingdom with a PUL.

## Results

A total of 120 Caucasian women (aged 18–45 years) with a PUL were recruited to the study. Patients' final pregnancy outcomes were classified according to the recent PUL consensus statement [12]. Final outcome definitions and details of the demographics of each group are provided in Table 1. There was no evidence of variation in age, weight or BMI between different final outcomes of PUL (one-way ANOVA).

**Table 1:** Patient recruitment: 120 patients with an initial diagnosis of a PUL were recruited to the study and grouped according to final pregnancy outcomes.

Group	Inclusion criteria	HCG (mU/ml)	Age (years)	Weight (Kg)	BMI	n
dVIUP	Definite viable intrauterine pregnancy: TVUSS confirmation of intrauterine gestational sac with yolk sac and embryo with cardiac activity.	6844±2017	28±1	70±4	26±2	28
dNVIUP	Definite nonviable intrauterine pregnancy: USS confirmation of intrauterine gestational sac with yolk sac and/or embryo without cardiac activity seen prior to uterine evacuation.	4022±1904	32±1	74±3	27±1	26
dEP	Definite ectopic pregnancy: intervention prompted by adnexal mass on TVUSS or by abnormal rise in serum hCG levels and confirmed at surgery and by histopathology.	1151±238	29±1	70±4	25±1	17
NP	Not pregnant: positive home pregnancy test result subsequently not confirmed by serum hCG measurement.	<5	26±2	70±8	27±3	11
srPUL	Spontaneously resolving PUL: PUL with spontaneous resolution of serum hCG levels.	428±114	32±1	74±4	28±1	27
tPUL	Treated persistent PUL: abnormal rise in serum hCG levels but no adnexal mass or IU sac seen on TVUSS after monitoring, managed medically with methotrexate.	400±188	32±4	83±15	28±5	3
pEP	Probable ectopic pregnancy: inhomogenous adnexal mass or extrauterine sac-like structure on TVUSS managed medically with methotrexate.	597±200	33±1	63±4	25±1	8

**Figure 1:**
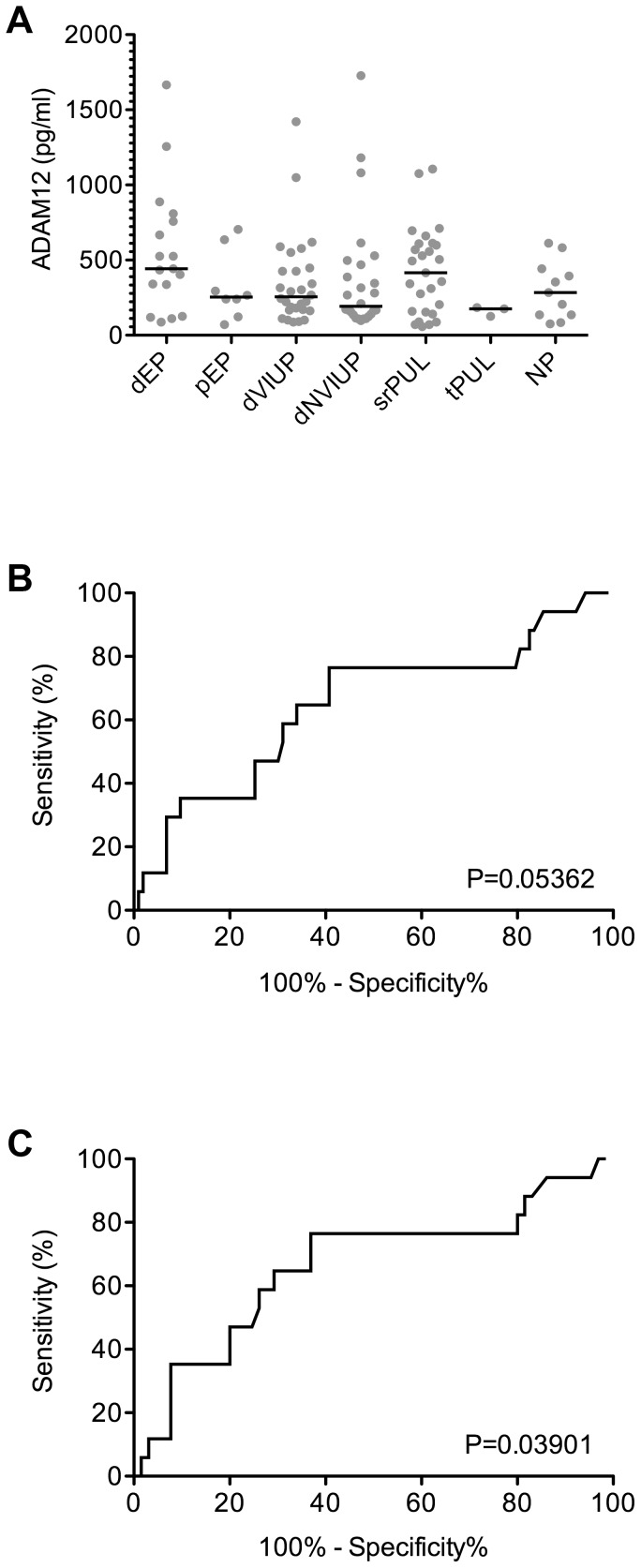
ADAM12 levels in sera collected from women at first presentation with a PUL, categorised according to final pregnancy outcome. Definite ectopic pregnancy (dEP: n = 17), probable ectopic pregnancy (pEP: n = 8), definite viable intrauterine pregnancy (dVIUP: n = 28), definite nonviable intrauterine pregnancy (dNVIUP: n = 26), spontaneously resolving PUL (srPUL: n = 27), treated persistent PUL (tpPUL: n = 3) and not pregnant (NP: n = 11). A ROC curve was generated (‘ROC of ADAM12’) to compare serum ADAM12 concentrations in patients with a dEP versus all other outcomes. The analysis was repeated (‘ROC of ADAM12 -PUL Data’) after ‘ambiguous’ pregnancy outcomes (srPUL, tpPUL and pEP) were excluded.

Serum ADAM-12 concentrations were elevated in patients with final outcomes of ‘definite ectopic pregnancy’ (dEP; median 442 pg/mL; 25% percentile 232 pg/mL, 75% percentile 783 pg/mL) when compared to: ‘definite viable intrauterine pregnancy’ (dVIUP; median 256 pg/mL; 25% percentile 168 pg/mL, 75% percentile 442 pg/mL); ‘definite non-viable intrauterine pregnancy’ (dNVIUP; median 192 pg/mL; 25% percentile 133 pg/mL, 75% percentile 476 pg/mL); ‘probable ectopic pregnancy’ (pEP; median 254 pg/mL; 25% percentile 152 pg/mL, 75% percentile 551 pg/mL); ‘treated probable PUL’ (tpPUL; median 177 pg/mL; 25% percentile 127 pg/mL, 75% percentile 184 pg/mL); or ‘non-pregnant women’ (NP; median 283 pg/mL; 25% percentile 137 pg/mL, 75% percentile 442 pg/mL) (Figure 1A). Serum ADAM-12 levels in patients with ‘spontaneously resolving PUL’ (srPUL; median 416 pg/mL; 25% percentile 154 pg/mL, 75% percentile 608 pg/mL) were similar to those in patients with dEP (Figure 1A).

When patients with ‘ambiguous’ PUL outcomes (srPUL, pEP and tpPUL) were included in the cohort for evaluation, ROC curve analysis indicated that ADAM-12 had little value as a diagnostic biomarker of EP (Area under ROC curve = 0.6465; P>0.05) (Figure 1B). However, when only well defined PUL outcomes (dEP, dVIUP, dNVIUP and NP) were included in the analysis, ADAM-12 appeared to have better diagnostic potential (Area under ROC curve = 0.6633; P<0.05) for detecting dEP (Figure 1C).

## Discussion

ADAM-12 may have some potential as a serum biomarker of dEP. However, we were unable to verify the findings of Rausch et al who had concluded ADAM-12 was a highly promising marker of ectopic pregnancy with strong diagnostic marker performance. In fact, we found that serum ADAM-12 concentrations in our UK cohort were elevated in patients with dEP compared to dVIUP (Figure 1A), rather than decreased as reported previously [8]. Furthermore, ADAM-12 did not appear to perform well as a biomarker of ectopic pregnancy.

We can only speculate why we were unable to replicate the promising findings described by Rausch et al [8]. They used a dissociation-enhanced lanthanide fluoroimmunoassay platform DELFIA/AutoDELFIA ADAM-12 research kit (PerkinElmer), compared to the ADAM12 Quantikine ELISA (R&D systems) used in the current study, and it is possible that the conflicting findings are due to this difference. However, it seems unlikely that this could account for the trend reversal observed between the two cohorts and differences in study design offer a more plausible explanation.

Gestational age is likely to be a key factor in ADAM12 levels, as it rises exponentially from around week 5 of the first trimester [13]. Therefore, it seems possible that the lower levels of ADAM12 we report reflect the gestational age of our prospectively collected first presentation cohort. Our study population was also slightly smaller (120 versus 199 participants) than that of Rausch et al. and we only recruited from a single UK center whereas Rausch et al. recruited from multiple US sites. This could potentially explain the disparity in our findings.

Another key difference in the design of our study and that published by Rausch et al. [8], is the inclusion of the entire range of PUL outcomes in the study design, not just dEP and dVIUP. Crucially, we found that serum ADAM-12 concentrations in patients who required surgical intervention for dEP were very similar to those observed in patients with final outcome of srPUL, who did not require surgical or medical intervention (Figure 1A).

Regardless of these differences, the discrepancy in the findings of our study and those of Rausch et al. [8] demonstrates the importance of verifying potential EP biomarkers in independent cohorts, and preferably from multiple international centers. The development of non-invasive blood biomarker test that reliably diagnoses EP remains an ongoing challenge.

## Materials and Methods

### Patient samples

Ethical approval for this prospective study was obtained from the Lothian Research Ethics Committee (LREC 04/S1103/20 and 09/S1103/39), with informed written consent obtained from all patients. Whole blood was obtained from women during their first clinical presentation with a positive home pregnancy test and abdominal pain and/or bleeding and a TVS that had been unable to locate the site of the pregnancy. After clotting for 2 hrs at RT, sera were collected and stored at −80°C in multiple aliquots. The women were monitored until their discharge from hospital and their final pregnancy outcomes were classified according to the recent PUL consensus statement [12].

### Ultrasound assessments

The ultrasound system used was the Toshiba Aplio XG and all of the ultrasound assessments were performed by a team of trained, qualified and experienced ultrasonographers.

### ADAM-12 ELISA

Sera were assayed using the ADAM12 Quantikine ELISA kit (R&D systems, Abingdon, UK) according to the manufacturers' instructions. Comprehensive details of the assay's performance parameters are available from the manufacturer (http://www.rndsystems.com/pdf/DAD120.pdf).

### Statistical Analysis

Statistical analyses, ELISA standard curve formulae and receiver operating characteristic (ROC) curves were generated using Prism 5.0 (GraphPad Software, La Jolla, USA).
